# Augmented cell survival in eutopic endometrium from women with endometriosis: Expression of c-myc, TGF-beta1 and bax genes

**DOI:** 10.1186/1477-7827-3-45

**Published:** 2005-09-08

**Authors:** M Cecilia Johnson, Marisa Torres, Alessandra Alves, Ketty Bacallao, Ariel Fuentes, Margarita Vega, M Angélica Boric

**Affiliations:** 1 Institute of Maternal and Child Research, School of Medicine, University of Chile; and San Borja Arriarán Clinical Hospital, Santiago, Chile

## Abstract

**Background:**

Endometriosis is a common gynaecological disorder characterized by the presence of endometrial tissue outside of the uterus. The fragments in normal menstruation are composed of necrotic and living cells, which do not survive in ectopic locations because of programmed cell death. The aim of this study was to evaluate if the balance between cell proliferation and apoptosis is changed in eutopic endometrium from women with endometriosis throughout the menstrual cycle by studying bax (pro-apoptotic), c-myc (regulator of cell cycle) and TGF-beta1 (involved in cell differentiation) genes.

**Methods:**

Eutopic endometrium was obtained from: 30 women with endometriosis (32.8 +/- 5 years) and 34 fertile eumenorrheic women (36 +/- 5.3 years). We analyzed apoptosis (TUNEL: DNA fragmentation); cell proliferation (immunohistochemistry (IHC) for Ki67); c-myc, bax and TGF-beta1 mRNA abundance (RT-PCR) and TGF-beta1 protein (IHC) in endometrial explants.

**Results:**

Cell proliferation strongly decreased from proliferative to late secretory phases in glands, but not in stroma, in both endometria. Positive staining in glands and stroma from proliferative endometrium with endometriosis was 1.9- and 2.2-fold higher than control endometrium, respectively (p < 0.05). Abundance of c-myc mRNA was 65% higher in proliferative endometrium from endometriosis than normal tissue (p < 0.05). TGF-beta1 (mRNA and protein) augmented during mid secretory phase in normal endometrium, effect not observed in endometrium with endometriosis. In normal endometrium, the percentage of apoptotic epithelial and stromal cells increased more than 30-fold during late secretory phase. In contrast, in endometrium from endometriosis, not only this increase was not observed, besides bax mRNA decreased 63% versus normal endometrium (p < 0.05). At once, in early secretory phase, apoptotic stromal cells increased 10-fold with a concomitant augment of bax mRNA abundance (42%) in endometria from endometriosis (p < 0.05).

**Conclusion:**

An altered expression of c-myc, TGF-beta1 and bax was observed in eutopic endometrium from endometriosis, suggesting its participation in the regulation of cell survival in this disease. The augmented cell viability in eutopic endometrium from these patients as a consequence of a reduction in cell death by apoptosis, and also an increase in cell proliferation indicates that this condition may facilitate the invasive feature of the endometrium.

## Introduction

Endometriosis is a common gynaecological disorder, frequently associated with infertility and pelvic pain that occurs almost exclusively in menstruating women of reproductive age. Although little is known about its aetiology and pathogenesis, the theory of retrograde menstruation and metaplasia of the mesothelium or peritoneum and implantation of viable endometrial cells has been widely accepted [1,2]. The morphology of eutopic endometrium from women with endometriosis is similar to normal endometrium, but its physiology and biochemistry are different. Recent reports show an abnormal survival capability at the epithelial and stromal levels of the eutopic endometrium from patients with endometriosis that may result in their continuous growth [3,4]

In proliferative phase, the endometrium is characterized by proliferation of cells from the basal layer that respond to 17β-estradiol, reaching its maximum at ovulation time and then, falling due to the effects of progesterone with marked changes in glandular epithelium and stroma. Besides steroids, other factors are involved in these processes. In fact, after the priming of estradiol, the expression of transforming growth factor beta1 (TGF-β1) is up regulated in human endometrium coincidently with the increase of plasma progesterone concentration, being down regulated during progesterone withdrawal. Mashburn et al. (1994) [5] reported that the highest concentration of this cytokine in the stroma was during the secretory phase and suggested that it may regulate epithelial cell proliferation and differentiation (for revision see Godkin and Dore, 1998) [6]. In epithelial cell lines, TGF-β plays a central role in maintaining the homeostasis through limitation of growth by repression of growth-promoting transcription factors, such as c-Myc [7]. As known, c-Myc is a transcription regulator that can both activate or inhibit gene expression in favour of cell proliferation.

It is well established that apoptosis is a temporary physiologic process by which tissues eliminate dysfunctional cells by a regulated mechanism that involves a sequence of intracellular molecular events including members of the Bcl-2 family, such as Bax, an inducer of apoptosis. In human endometrium, Bax is expressed differentially throughout the menstrual cycle, with an expression mainly in late phase according to the number of apoptotic cells detected at the end of the menstrual cycle [8,9].

The aim of the present study was to evaluate whether the balance between cell proliferation and apoptosis is changed in eutopic endometrium from women with endometriosis throughout the menstrual cycle. For this purpose, we evaluated: cell proliferation by the detection of Ki67 and c-myc mRNA abundance, apoptosis by DNA fragmentation detection and bax mRNA abundance, and the expression of TGF-β1 in eutopic endometrium obtained from women with and without endometriosis.

## Materials and methods

### Subjects

Eutopic endometrial tissue was obtained from 30 women undergoing laparatomy for endometriosis associated with chronic pelvic pain, severe dysmenorrhea and infertility, and from 34 eumenorrheic women undergoing laparoscopy for tubal sterilization. The age of these women was 32.8 ± 5 years; range 23 to 40 years and 36 ± 5.3 years; range 25 to 43 years, respectively (p < 0.05). Both groups of patients had normal body mass index (BMI ≤ 25) and they were without hormonal treatment and at least 3 months without contraceptive pills before surgery. The endometrial samples were obtained with a Pipelle suction curette from the corpus of the uterus and washed several times with ice-cold phosphate buffered saline (PBS) to remove blood. Each patient signed a written informed consent and the Institutional Ethic Committee approved this study.

The endometria were dated according to established criteria [10] by an experienced histopathologist and classified as proliferative (days 1–14, women without (n = 10) or with (n = 8) endometriosis) or secretory endometria (days 15–28, women without (n = 24) or with (n = 22) endometriosis). For both groups of endometria, the secretory phase was divided in early (days 15–18, n = 15), mid (days 19–23, n = 18) and late (days 24–28, n = 13). Once obtained, the tissue was cut into slices and some pieces were immediately frozen in liquid nitrogen for mRNA preparation or placed in 4% formalin/PBS (pH 7.2–7.4) for histological evaluation, cell death determination and immunohistochemistry.

#### Immunohistochemistry of Ki67 and TGF-β1

Sections (4 to 6 μm thick) of human endometrial tissue at different stages of the menstrual cycle were deparaffinized in xylol and hydrated gradually through graded alcohols. The sections for Ki67 detection were incubated in 10 mmol/L citrate buffer (pH 6.0) at 98°C for 20 min. All slides were quenched in 3% H_2_O_2 _for 5 min at room temperature, then blocked with 2% BSA in PBS (w/v) for 1 h at room temperature. Primary antibodies for Ki67 (1:75, Dako, Carpinteria, LA) and TGF-β1 (1:1000, CBL 778 clon TB21; Cymbus Biotechnology LTD, Hamts, NF) were applied to the samples and incubated at 37°C for 2 or 1 h, respectively. Immunodetection was performed by using the streptavidin-biotin peroxidase system (LSAB 2, Dako), diaminobenzidine (DAB) as chromogen and counterstained with hematoxylin. The immunohistochemical evaluation of Ki67 and TGF-β1 was determined as the percentage of positive stained cells. In all cases, three blinded observers evaluated at least 1,000 cells in one section from each sample.

#### RNA preparation, cDNA synthesis, Reverse Transcription-Polymerase Chain Reaction

Total RNA was isolated from frozen proliferative and secretory endometria using RNA-solv Reagent (Omega Bio-teck, Lilburn, GA) plus glycogen (Chemicon International, Inc., Temecula, CA); then the purified pellet was resuspended in diethylpyrocarbonate-treated water. Complementary DNA (cDNA) was synthesized from 2 μg of total RNA digested previously by DNase I (Fermentas AB, Vilnius, Lithuania) using random primers (Invitrogen, Carlsbad, CA) and 200 U revertAid H Minus M-MuLV reverse transcriptase (Fermentas) following the manufacturer's instructions.

The amplification of bax mRNA was performed as indicated in Johnson et al. (2004a) [11]; c-myc, and TGF-β1 mRNAs were studied using specific pairs of primers: c-myc (330 bp), NID: g34815, upstream 5'-GAT TCT CTG CTC TCC TCG A-3' and downstream 5'-CTC TGA CAC TGT CCA ACT TG-3'; TGF-β1 (307-bp), NID:gi 10863872, upstream 5'-CAC CAA CTA TTG CTT CAG C-3' and downstream 5'-GAT CAT GTT GGA CAG CTG-3'. Two μl of cDNA per reaction were adjusted to a total volume of 25 μL by adding PCR buffer containing 3 mmol/L MgCl_2_, 0.125 U of Taq DNA polymerase (Invitrogen), 0.25 mmol/L nucleotide mix and 0.4 μmol/L of each specific human primer (Invitrogen). As internal control, 18S rRNA cDNA [12] was amplified in each sample in the same conditions described above. The reaction was performed in ThermalCycler PT-100 (MJ Research Inc. Watertown, MA) at denaturation: 94°C for 45 sec; annealing: 55°C for 60 sec for c-myc, bax and 18S rRNA, or 53°C for TGF-β1; extension: 72°C for 60 sec, and repeat for 18 cycles for 18S rRNA, 30 cycles for c-myc and 33 cycles for bax and TGF-β1. To determine that the amplification of all the genes were within a linear range, we previously evaluated the linearity of amplification of the corresponding transcripts in human endometria explants and the number of cycles were then decided.

Amplified fragment products were visualized on a 1.0% agarose gel using ethidium bromide staining. Semiquantification of PCR-products was performed by image analysis (Kodak EDAS 290 Electrophoresis Documentation and Analysis System, Kodak 1D Image Analysis Software, Rochester, NY), and its identity was confirmed by sequencing (ABI PRISM model 310 version 3.4 automatic sequencer; Perkin Elemer cetus, Norwak, CT).

#### Apoptosis detection system

DNA fragmentation was assessed by TUNEL according to the specifications of the manufacturer and adapted by us [13]. Briefly, paraffin tissue sections mounted on silane-coated slides (4–6 μm thick) were deparaffinized with xylol, rehydrated and fixed with 4% methanol-free formaldehyde/PBS; then deproteinated for 10 min (20 μg/mL proteinase K in PBS), washed and post-fixed again. To avoid artificial DNA fragmentation, only nuclease-free solutions were used during the whole procedure.

The labeling of 3'-OH ends of fragmented DNA was performed by incubation with fluorescein-12-deoxy-UTP using 12.5 U terminal deoxynucleotidyl transferase (TdT) for 1 h at 37°C in a humidified chamber. As positive control, normal endometrium pre-treated with DNase displayed nuclear positive reactivity in the majority of cells, demonstrating the incorporation of the fluorescent nucleotide at 3'-OH ends to the fragmented DNA. To estimate non-specific binding and autofluorescence, negative controls were included in all assays where tissue sections were incubated without the TdT enzyme. Slides were analyzed by fluorescence microscopy with a wide-band excitation barrier filter suitable for analyzing both green (fluorescein labelled fragmented DNA) and red (propidium iodide counterstain) fluorescence. Three independent observers selected different optical fields in a random manner to determine the percentage of positive staining in at least 1,000 cells for each sample. The assessment of DNA fragmentation by the TUNEL analysis has been shown to detect mainly apoptosis, although positive staining in necrotic cells cannot be ruled out. Therefore, in this study we confirmed the presence of morphological apoptosis by light microscopy and the absence of necrosis was established by morphological criteria.

### Statistical analysis

Results were expressed as mean ± SEM of the number of experiments indicated in the figure legends. The age of the women was expressed as mean ± SD. Data were statistically analyzed by Student's t-test (age women) or one-way ANOVA followed by Tukey's Multiple Comparison test (Tables and Figures). Difference was considered statistical significant at p less than 0.05.

## Results

### Cell proliferation

**Figure 1:**
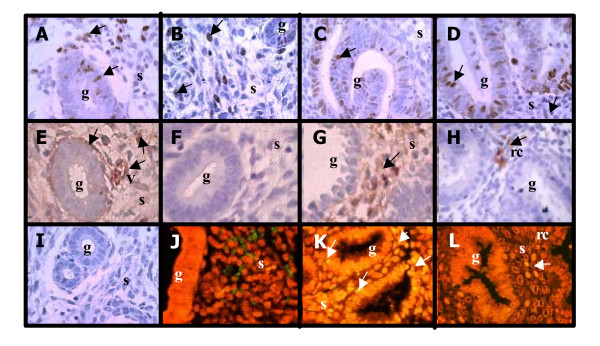
Immunohistochemical staining for Ki67, TGF-β1 and apoptosis by TUNEL in explants of human endometrium throughout the menstrual cycle obtained from normal (A, C, E, G, I, J and K) and endometriosis (B, D, F, H and L) women. Representative human endometrium explants from (A, B, E, F and I) proliferative phase and (C, D, G, H, J, K and L) secretory phase of the menstrual cycle with positive immuno-staining for (A-D) Ki67 and (E-H) TGF-β1 or positive DNA fragmentation by TUNEL (K and L). Immunohistochemitry (I) and TUNEL (J) negative controls. Cell nuclei are stained with haematoxylin (immunohistochemitry) or propidium iodine (TUNEL). g: glandular, s: stroma, rc: red blood cell. Magnification, 400×.

Nuclear immunostaining of Ki67 was detected in the cell nucleus of both epithelial and stromal compartments during the menstrual cycle in eutopic endometrium of women with or without endometriosis (Figure 1A–D). Cell proliferation decreased from proliferative to mid and late secretory phases in the normal epithelial gland of both endometria (p < 0.05), with no important changes in the stromal compartment (Table 1). The positive staining observed in epithelial and stromal proliferative endometrium obtained from women with endometriosis was 1.9- and 2.2-fold higher than normal proliferative endometrium, respectively (p < 0.05). A tendency of higher cell proliferation was observed in epithelium from endometriosis than in normal epithelium obtained during early and mid secretory phases (Table 1).

**Table 1:** Percentage of Ki67 in human eutopic endometrium obtained from women without and with endometriosis during the menstrual cycle.

	Normal		Endometriosis	
	
	Gland	Stroma	Gland	Stroma
Proliferative	12.9 ± 4.2	7.7 ± 2.4	27.3 ± 5.3*	16.9 ± 3.8*
Secretory Early	12.4 ± 5.7	5.3 ± 0.6	26.1 ± 5.2	9.3 ± 2.0
Secretory Mid	0.8 ± 0.1^# °^	4.3 ± 0.8	10.8 ± 5.7^°^	5.7 ± 1.6^#^
Secretory Late	1.0 ± 0.7^# °^	10.9 ± 2.4	1.3 ± 0.9^# °^	10.5 ± 3.0

Percentage of positive cells was performed in eutopic endometrium explants obtained from women without (normal) and with endometriosis at different stages of the menstrual cycle as indicated in Material and Methods. Results are given as mean ± SEM from: 8 and 7 endometria without and with endometriosis in each stage, respectively. *p < 0.05 vs. normal endometrium. ^#^p < 0.05 vs. proliferative phase. ^°^p < 0.05 vs. early secretory phase.

### Detection of TGF-β1 and *c-myc *in eutopic endometrium obtained from women with and without endometriosis during the menstrual cycle

During the menstrual cycle, the mRNA of *TGF-β1 *increased more than 50% in mid and late secretory phases in normal endometrium (p < 0.05), effect that was not detected in eutopic endometrium obtained from women with endometriosis, remaining unchanged throughout the cycle (Table 2).

**Table 2:** *TGF-β1 *amplification from endometrium cDNA of women without and with endometriosis during the menstrual cycle.

	Normal	Endometriosis
Proliferative	1.12 ± 0.1	0.92 ± 0.2
Secretory Early	0.82 ± 0.2	0.86 ± 0.1
Secretory Mid	1.56 ± 0.2^#^	1.10 ± 0.1*
Secretory Late	1.49 ± 0.1^#^	1.05 ± 0.1*

Data correspond to amplification rate relative to *18S rRNA*. Results are given as mean ± SEM from: 8 and 5 proliferative; 7 and 8 early; 4 and 8 mid and 4 late secretory endometria obtained from women without (normal) and with endometriosis, respectively. *p < 0.05 vs. normal endometrium; ^#^p < 0.05 vs. early secretory phase.

Positive TGF-β1 immunostaining was homogenously distributed in the cytoplasm of stromal cells, and a weak intensity was observed in glands of both types of endometrium (Figure 1E–H). The cytokine was also detected as secretion in the glands lumen and extracellular matrix, besides an intense brown staining in blood vessels and red blood cells (1E and 1H). In normal endometrium, the highest percentage of TGF-β1 positive stromal cell was 53 ± 9% at mid secretory phase with an increase by 2-fold compared to proliferative phase (p < 0.05), whereas no differences were observed in epithelial cells. In eutopic endometrium from endometriosis, the percentage of TGF-β1 positive proliferative stromal cells was similar to normal proliferative stromal cells (28.18 ± 10.6 and 27.3 ± 6.5, respectively) and did not change during the menstrual cycle. It was almost undetected in eutopic endometrium gland from endometriosis.

**Figure 2:**
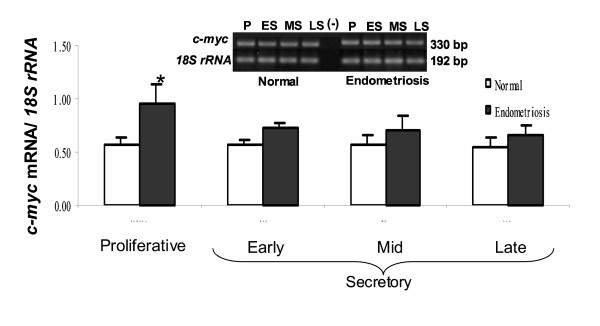
PCR amplification from endometrium cDNA of women without (Normal) and with endometriosis using primers for *c-myc *(330-bp) and *18S rRNA *(192-bp). Representative gel is shown. Graph illustrates the corresponding amplification relative to *18S rRNA *and the results are given as mean ± SEM from: 6 and 6 proliferative (P); 6 and 5 early secretory (ES); 5 and 4 mid secretory (MS) and 5 and 4 late secretory (LS) eutopic endometria obtained from women without and with endometriosis, respectively. (-) PCR amplification without template. *p < 0.05 vs. normal endometria.

The abundance of *c-m*yc mRNA was not significantly changed throughout the menstrual cycle in both types of endometrium; however, the proto-oncogen was 65% higher in proliferative endometrium with endometriosis than normal endometrium, respectively (p < 0.05) (Figure 2).

### Cell death by apoptosis

**Figure 3:**
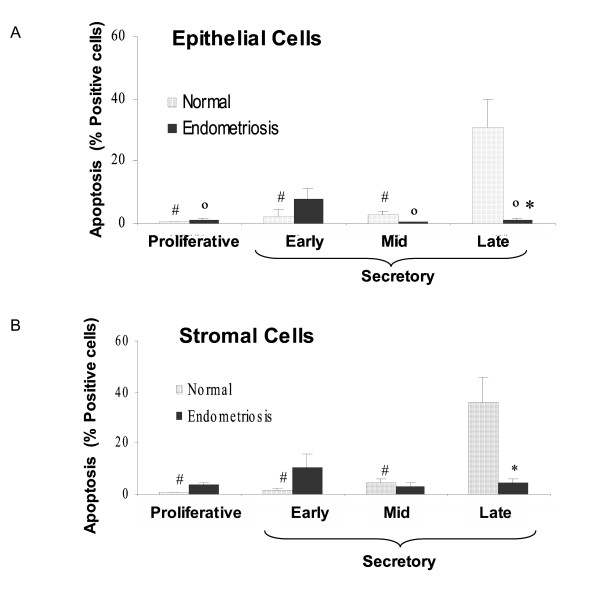
Percentage of apoptotic cells in epithelial (A) and stromal cell (B) compartments in human endometrium explants obtained from normal women and women with endometriosis. Histological sections were evaluated by TUNEL technique (see text). The results are given as mean ± SEM from: 5 and 6 proliferative; 5 and 6 early secretory; 4 and 5 mid secretory; 7 and 6 late secretory eutopic endometria obtained from women without (normal) and with endometriosis, respectively. *p < 0.05 vs. normal endometria. ^°^p < 0.05 vs. early secretory phase. ^#^p < 0.05 vs. late secretory phase.

DNA fragmentation was strongly detected by TUNEL mostly in epithelial and stromal cells in normal endometrium from late secretory phase (Figure 1K), effect that was not observed in late endometrium from women with endometriosis (Figure 1L). The percentage of apoptotic cells in late normal endometrium increase more than 30-fold in both cell compartments compared to proliferative endometrium (p < 0.05) (Figure 3A and 3B). In proliferative, early and mid secretory endometria, a reduced number of positive apoptotic cells were detectable in both groups of women. Interestingly, in early secretory endometrium from women with endometriosis, the number of apoptotic cells was increased respect to normal endometrium by 10-fold in stromal cells (p < 0.05) (Figure 3A and 3B).

**Figure 4:**
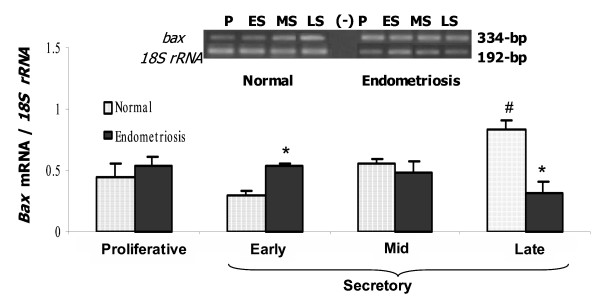
PCR amplification from endometrium cDNA of women without (Normal) and with endometriosis using primers for *bax *(334-bp) and *18S rRNA *(192-bp). Representative gel is shown. Graph illustrates the corresponding amplification relative to *18S rRNA *(192-bp) and the results are given as mean ± SEM from 5 and 5 proliferative (P); 6 and 5 early secretory (ES); 5 and 6 mid secretory (MS) and 4 late secretory (LS) eutopic endometria obtained from women without (normal) and with endometriosis, respectively. (-) PCR amplification without template. *p < 0.05 vs. normal endometria. ^#^p < 0.05 vs. proliferative phase.

The pro-apoptotic gene *bax *was amplified in both groups of endometrium (Figure 4). The abundance of *bax *mRNA increased at the end of the menstrual cycle in normal endometrium. Although no significant difference was observed in proliferative and mid secretory phases, *bax *mRNA increased 42% in early and reduced 63% in late secretory endometria from women with endometriosis respect to normal endometrium, respectively (p < 0.05) coincident to the results obtained for apoptotic index.

## Discussion

It is known that eutopic endometrium from women with endometriosis presents physiological differences with normal endometrium. In this study we show that eutopic endometrium from these patients has a significant increase in cell survival, and alterations on DNA fragmentation and on the expression of factors involved in cell proliferation, cell differentiation and programmed cell death induction, such as Ki67, *c-myc*, TGF-β1 and *bax*.

In agreement with previous reports and in accordance with results of light microscopy [10,14,15], our data show that epithelial cells from normal endometrium exhibit a higher proliferation rate than stromal cells during proliferative and early secretory phases, stages of the menstrual cycle regulated mainly by estradiol. Although eutopic endometrium from patients with endometriosis present a similar Ki67 distribution throughout the menstrual cycle, a significant augment of cells into cell cycle compared to normal endometrium was observed, in accordance with other investigators [16-18]. Even though Ki67 is expressed in the cell during M, G1, S and G2 phases of cell cycle and is absent in resting cells (Go), a good correlation between Ki67 and mitotic indices has been reported in human endometrium [19]. In contrast, other groups [4,20] have reported no differences between normal and eutopic endometria from women with endometriosis, probably due to the use of different techniques and the detection of proliferating cell nuclear antigen (PCNA), a nuclear protein restricted to S phase.

Several genes involved in cell proliferation, such as the proto-oncogene *c-myc *are up-regulated by estradiol in different cell types that include human breast cancer cell line MCF-7 [21,22], rat hepatocyte [23], and pituitary and somatolactotrophic cell line GH3 [24]. Little information is available of the proto-oncogen *c-myc *in the endometrium. The presence of the protein c-Myc in nuclei and cytoplasm of endometrial glands and nuclei stroma from eutopic and ectopic endometria has been reported [25], even though its role in the endometrium is not clear. In the present investigation, the *c-myc *mRNA abundance was not modified throughout the menstrual cycle in both types of endometria studied, although it was augmented in eutopic endometrium from women with endometriosis compared to normal women. Several findings suggest that in endometriosis, ectopic endometrium grows and regresses in an estrogen-dependent manner. Even more, eutopic endometrium of women with endometriosis contains not only estradiol receptors, but also P_450_Arom, the enzyme that catalyzes the conversion of androgen to estrogen, provoking an estrogenic micro-environment in this tissue that may act through intracrine pathways [26-28]. Therefore, the estrogenic microenvironment could induce the expression of the transcription factor *c-myc *and facilitates cell proliferation in the eutopic endometria in this disease. This effect may be improved by the reduction of TGF-β1 detected in the endometrium of these patients, as probable consequence of the loss of the negative regulation on P_450_AROM described in choriocarcinoma cell line (JEG-3) [29].

On the other hand, TGF-βs are likely candidates to partially mediate the effects of progesterone on the expression of other proteins, which have been suggested to be involved in the regulation of endometrial cell proliferation and differentiation [5,6]. In addition, the cyclic expression of TGF-β1, mainly observed in stromal cells during the secretory phase as reported in this study, is in accord to progesterone receptor location [30]. In several cell types, TGF-β1 plays a role in maintaining tissue homeostasis by modulating genes involved in the arresting of cell cycle including down-regulation of c-Myc [7,31]. Even more, in rabbit uterine epithelial cells, a coordinated but inverse regulation of cell proliferation and apoptosis by this cytokine was described [32]. The fact that the cyclicity of TGF-β1 observed in normal endometrium was absent in eutopic endometrium from women with endometriosis indicates an impaired local production in these patients that may favors the cell proliferation. More studies are required to understand the complex and strict interactions between these molecules involved in the deregulation on cell proliferation in the eutopic endometrium of these women.

Proliferation and apoptosis are important biologic processes in the endometrium remodeling with cell cycle changes that take place during the menstrual cycle [14,33]. In the late secretory phase, and related with a decrease of endometrial progesterone receptor and plasma progesterone concentration, cell death by apoptosis increases in the functional endometrial layer, and at the time of menstruation it becomes necrotic and hypoxic and is shed. In the present investigation, the expected increase of apoptotic cells during late secretory phase was not observed in eutopic endometrium from women with endometriosis. Concomitantly to the absence of apoptosis in those endometria, the pro-apoptotic gene *bax *mRNA abundance was unchanged during the menstrual cycle, at difference at the increase exhibited in normal endometrium, suggesting that the reduction of bax expression may be a mechanism that could explain the decreased incidence of apoptosis in endometriosis. In agreement with these results, recent publications [3,34,35] also reported the absence of apoptotic cells and the increase of Bcl-2 and the reduction of Bax expression in eutopic endometrium from women with endometriosis, although Béliard et al. (2004) [4] were unable to find these differences between patients with endometriosis and normal women. Curiously, we detected that both *bax *mRNA abundance and apoptotic cells were increased in early secretory phase in eutopic endometrium from women with endometriosis but not from normal women. The implication of this augmented cell death in eutopic endometrium from the patients needs more studies.

It is well known that menstrual fragments are composed of both necrotic and living cells, which do not survive in ectopic locations because of its programmed cell death [36,37]. The loss of cell death by apoptosis in late secretory phase in these patients seems to be consistent with cell ectopic survival and implantation of endometrial cells in the peritoneal cavity, effect that is independent of the disease stages as it was reported by Dmowski et al. (2001) [34]. The altered expression of *c-myc*, TGF-β1 and *bax *observed in eutopic endometrium from women with endometriosis, suggests their participation in the deregulation of the cell survival in this tissue. It is known that the deregulation of apoptotic signaling can play a primary or secondary role in various diseases; in fact, an insufficient apoptosis degree contributes to pathogenic processes including cancer. The present investigation shows that cell viability is augmented in eutopic endometrium from women with endometriosis compared to normal endometrium, primarily due to a reduction in cell death by apoptosis, and also, an increase in cell proliferation. These data strongly suggest that this condition may facilitate the invasive character of the endometrium in these patients. Until now, it is not clear why misplaced endometrial cells from healthy women do not implant and do not develop into endometriotic lesions as occurs in women that develop endometriosis, indicating that some factor(s) which facilitate(s) their survival and implantation may be involved. However, we cannot rule out the possible presence of endometrium cells that belong to basal layer, aspect not studied in the present work.

## Conclusion

The altered expression of *c-myc*, TGF-β1 and *bax *observed in eutopic endometrium from women with endometriosis, suggests the participation of these molecules in the regulation of the cell survival in this disease. The augmented cell viability in eutopic endometrium from these patients as consequence of a reduction in cell death by apoptosis, and also an increase in cell proliferation indicates that this condition may facilitate the invasive character of the endometrium. The estrogenic microenvironment reported in this tissue in women with endometriosis may explain this improvement of proliferation/apoptosis balance.

## Contribution of the authors

MCJ conceived and designed the study, participated in the analysis and interpretation of data, and drafted the manuscript. MT carried out the Ki67 IHQ and TUNEL, participated in the analysis of data. AA carried out TGF-β1 IHQ and participated in analysis of data. KB carried out the mRNA studies and participated in the analysis of data, AF carried out the statistical analysis and helped to draft the article, MV participated revising the manuscript critically and helped to draft the article, MAB participated in the analysis and interpretation of data and helped to draft the manuscript. MT, AA and KB performed the microscopic evaluation.
